# Control Work

**Published:** 1900-05

**Authors:** 


### CONTROL WORK.

North Carolina now demands a tuberculin-test certificate of all cattle entering the State for breeding or dairy purposes. Said certificate must contain the signatures of the sanitary authorities and State veterinarian of the State from which the animal or animals come. Transportation companies are directed not to deliver live-stock for any purpose but immediate slaughter without certificate.

Certificates showing a freedom from tuberculosis are now demanded for all cattle entering for breeding or dairy purposes into Connecticut (physical test), Canada, Illinois, Kansas, Massachusetts, Maine, Montana, Missouri, Nebraska, New Hampshire, New Jersey, New Mexico, New York City, North Carolina, Pennsylvania, Rhode Island, Vermont, and Texas.

The division of Animal Pathology of the Nebraska Experiment Station of the University of Nebraska has been busy since the first of the year in the distribution of the blackleg vaccine, in which it is co-operating with the Bureau of Animal Industry of the United States Department of Agriculture. The increase in the distribution is shown in the fact that during the year 1899 the division furnished blackleg vaccine to 203 applicants in fifty-six counties, the total number of doses being 23,256. Since January 1, 1900, the division has furnished the vaccine to 211 applicants in forty-seven counties, the number of doses aggregating 15,000. In the corresponding period of 1899 twenty-three applicants received 1500 doses, or one-tenth as much as the present season. This greater demand for vaccine is due rather to an increased knowledge of and confidence in the remedy than to an increase in the prevalence of the disease.

The records show that the calls have come from persons that used the vaccine last year and from their neighbors.

The reports of the applicants show that it has reduced the mortality, absolute satisfaction having been given in over 96 per cent. of the cases.

This work is being taken up very enthusiastically by farmers and stockmen of the State. It has been one of the very important subjects that have been discussed at Farmers' Institutes by this division of the station. The division at these institutes explained the process of the manufacture of the vaccine and how it is applied. There is now ready for publication by the station a bulletin which treats on the subject in a popular way, dealing with the age and species of animals attacked, the cause of the disease, disposition of the blackleg carcass, symptoms, post-mortem appearances, methods of treatment, and a full discussion of what is regarded as the only reliable and economical method, vaccination.

The division of Animal Pathology of the Experiment Station at the University of Nebraska is perfecting its plans for the coming year's campaign against hog-cholera. As is well-known, this division has been very active in inaugurating experiments with this disease. One of the experiments under contemplation is the taking up of certain counties in which hog-cholera exists and having experts there to note the mortality in the entire county, and to aid the officials of the county in eradicating the disease both by sanitary and popular methods and by the use of serum. It is hoped that if certain counties will take up this idea of assisting this division a great deal of good can be done, both in ascertaining how virulent the disease has been in the county and by reducing the percentage of mortality in the county. In other words, it is a kind of quarantine system, or stamping-out process ; and it is hoped by this method that farmers can guard against this disease to a large extent. The plans are not as yet fully matured, but will be perfected before the work is begun.

North Carolina has placed herself thoroughly in accord with the General Government looking to the control and eradication of Texas fever among cattle. The establishing of quarantine lines, the movements of all cattle in infected territory, and the regulation of all transportation centres and lines are to be in accord with Federal regulations, subject to all modifications as conditions arising may necessitate.

The veterinary profession of New York State could not do better than urge strongly among the stock-owners, breeders, and their clients and others the adoption of the plans now in force in Pennsylvania for the better control and suppression of tuberculosis.

Its condemnation of the methods pursued by those directing the Farmers' Institutes in employing members of the profession with untenable views and ignorant of the progress of science in the control and eradication of this disease continues to menace good work in this direction.

Montana's regulations for the control and eradication of sheep scab will do much for the people of that State if the latter will coöperate with the district inspectors. State Veterinarian Knowles in his bulletin covers the subject in a thorough manner and points the way of relief from this animal pest.

